# CSF cytokine, chemokine and injury biomarker profile of glial fibrillary acidic protein (GFAP) autoimmunity

**DOI:** 10.1002/acn3.52305

**Published:** 2025-01-27

**Authors:** Yahel Segal, Georgios Mangioris, Vanda Lennon, Binxia Yang, Divyanshu Dubey, Eoin P. Flanagan, Andrew McKeon, John R. Mills, Michel Toledano, Ivana Vodopivec, Sean J. Pittock, Anastasia Zekeridou

**Affiliations:** ^1^ Department of Laboratory Medicine and Pathology Mayo Clinic Rochester Minnesota USA; ^2^ Department of Neurology Mayo Clinic Rochester Minnesota USA; ^3^ Center for MS and Autoimmune Neurology Mayo Clinic Rochester Minnesota USA; ^4^ Roche Product Development—Neuroscience F. Hoffmann‐La Roche Ltd Basel Switzerland

## Abstract

Defining the CSF cytokine/chemokine and injury biomarker signature of glial fibrillary acidic protein (GFAP) autoimmunity can inform immunopathogenesis. CSF GFAP‐IgG‐positive samples (*N* = 98) were tested for 17 cytokines/chemokines, neurofilament light chain (NfL), and GFAP (ELLA, Bio‐Techne). Controls included non‐inflammatory (*N* = 42), AQP4‐IgG‐positive (*N* = 83), CNS infections (*N* = 13), and neurosarcoidosis (*N* = 32). IL5, IL6, IL10, IL8/CXCL8, CXCL9, CXCL10, CXCL13, BAFF, GM‐CSF, IFN‐gamma, and TNF‐alpha concentrations were higher compared to non‐inflammatory controls (*P* < 0.01). GFAP concentrations were similar to those of AQP4‐IgG‐positive patients; NfL was higher (*P* < 0.001) and correlated with MRI changes and outcomes. CSF cytokine/chemokine findings in GFAP autoimmunity correlate with histopathology; GFAP and NfL hold promise as disease biomarkers.

## Introduction

Glial fibrillary acidic protein (GFAP) immunoglobulin G (IgG) is one of the most common neural autoantibodies in patients with central nervous system (CNS) autoimmunity and a biomarker of a steroid‐responsive subacute meningoencephalomyelitis with or without papillitis.^1^, ^2^, ^3^ The pathophysiology of GFAP autoimmunity is not fully elucidated. A murine model of GFAP‐specific CD8^+^ T‐cell autoimmunity revealed a spontaneous chronic relapsing–remitting course and a viral‐induced acute, monophasic course.^4^ Necrotizing inflammation has been described in pug dogs with GFAP autoimmunity, while human histopathological studies revealed a lymphocytic and a granulomatous phenotype, potentially representing distinct pathomechanisms or disease stages.^5^

Previous studies reporting elevated cerebrospinal fluid (CSF) levels of tumor necrosis factor alpha (TNFα), interleukin (IL)27, IL6, CCL20,^6^ IL1‐beta, and IL17^7^ in patients with GFAP autoimmunity were limited by size and by the inclusion of patients without CSF GFAP‐IgG positivity.^2^, ^3^ In this study, we evaluated CSF cytokines/chemokines and neural injury markers of GFAP‐IgG CSF‐positive patients.

## Methods

This retrospective cohort study was approved by the Mayo Clinic Institutional Review Board (IRB # 21‐000918); patients gave consent for their records to be used for research.

All CSF GFAP‐IgG‐positive patients (tissue‐ and cell‐based indirect immunofluorescence confirmed) from the Mayo Clinic Neuroimmunology Laboratory (07/2023–01/2024) and all historical GFAP‐IgG CSF‐positive patients with available clinical information and CSF were included (*N* = 98 total, 56 with clinical data). Patients were excluded if they had coexisting neural autoantibodies, systemic inflammatory disease, CNS malignancy, or CNS infection. Clinical samples were collected either prior to immunotherapy administration (17/56, 30%); following immunotherapy administration (10/56, 18%); or lacking data on the exact date of immunotherapy administration (29/56, 52%). Clinical data were obtained through electronic chart review or communication with treating physicians; poor outcome was defined as a modified Rankin Score (mRS) >2 or the need for bilateral gait assistance. Patients were considered atypical in the absence of meningoencephalitis/papillitis along with nonconsistent ancillary findings.^3^ Control cohorts (Table S1) included patients with noninflammatory disorders (*N* = 42), viral meningoencephalitis with microbiologic confirmation (*N* = 13), aquaporin‐4 (AQP4)‐IgG‐positive CSFs (without clinical information, previously reported,^8^ *N* = 83), and neurosarcoidosis patients (previously reported,^8^ *N* = 32).

We assessed CSF IL1‐beta, IL2, IL4, IL5, IL6, IL10, IL12p70, IL13, IL17A, IL8/CXCL8, CXCL9, CXCL10, CXCL13, BAFF, GM‐CSF, IFN‐gamma, and TNF‐alpha using a multiplexed automated immunoassay (ELLA by Bio‐Techne), recently validated for CSF testing^8^; CSF GFAP and NfL concentrations were also evaluated in all GFAP‐IgG‐positive and in AQP4‐IgG‐positive patients with remaining CSF (*N* = 48 for GFAP levels, *N* = 83 for NfL levels). Analyte concentrations were measured in duplicate or triplicate. Values were excluded (indicated as missing) if there was a >20% difference between duplicates/triplicates within the reported linear range.

Cytokine/chemokine analyte concentrations were considered elevated if exceeding the maximum value of non‐inflammatory controls or the lower limit of quantification (LLOQ) for the assay, whichever was higher. In order to focus on analytes that were elevated across a substantial number of patients and thus more clinically relevant, only analytes with abnormal values in ≥20% of GFAP‐IgG‐positive patients were considered elevated and compared to other inflammatory group controls. Groups were compared with the Wilcoxon rank sum test or Fisher's exact test, as appropriate. Values of *P* < 0.05 were considered significant. Missing values were excluded. The statistical software R (The R Foundation for Statistical Computing, Vienna, Austria) was used.

## Results

### Clinical characteristics

The cohorts' baseline characteristics are summarized in Table S1; sex and age differences are consistent with the underlying diagnoses. Ninety‐eight CSF GFAP‐IgG‐positive patients were included with a median age of 43 years; 52% were female. Clinical data were available for 56 patients (Table 1). The most common clinical phenotypes were meningoencephalitis with or without myelitis; nine patients had atypical presentations. MRI changes attributed to GFAP autoimmunity were present in 79%. CSF pleocytosis was present in 82% (median 119 cells/mm^3^, range 0–1,978); 55% of CSFs with data available (16/29) were collected within 1 month of symptom onset and 30% (17/56) were known to be immunotherapy‐naïve at collection. Most patients (44/52, 85%) received immunotherapy during their disease course; poor outcomes were noted in 38% (10/26).

**Table 1:** Clinical characteristics of patients with GFAP autoimmunity (*N* = 56).

Clinical syndrome	
Meningoencephalitis (%)	31/56 (55)
Meningoencephalomyelitis (%)	22/56 (39)
Papillitis (%)	2/56 (4)
Isolated myelitis (%)	1/56 (2)
Flu‐like symptoms^a^ (%)	19/55 (35)
Clinical signs of meningeal involvement (%)	19/53 (36)
Clinical myeloradiculitis (%)	17/55 (31)
Atypical presentation^b^ (%)	9/56 (16)
MRI	
Any disease‐related imaging abnormality (%)	44/56 (79)
Spinal cord involvement (%)	18/24 (75)
Gadolinium enhancement (%)	33/54 (61)
CSF	
Collected within 1 month of symptom onset (%)	16/29 (55)
Elevated CSF WBC (%)	45/55 (82)
WBC, median (range), per mm^3^	119 (0–1,978)
Protein, median (range), mg/dL	70 (8–326)
High CSF GFAP IgG titer^c^ (%)	54/85 (64)
Treatment	
Received IST at any time point (%)^d^	44/52 (85)
Received IST at/prior to CSF collection (%)	10/27 (37)
Outcome	
Poor outcome^e^ (%)	10/26 (38)
Median follow‐up duration (*N*, range)	16 (13, 1.5–84)

CSF, cerebrospinal fluid; IST, immunosuppressive therapy; mRS, modified Rankin score; WBC, white blood cells.

^a^ Any viral‐like symptoms reported within 2 weeks prior to the onset of CNS symptoms, or concurrent with initial CNS symptoms.

^b^ Patients who presented with symptoms other than meningitis/encephalitis/myelitis/papillitis and with at least two of: normal CSF or imaging or an insidious course of symptom development (>12 months).

^c^ High titer was defined as >1:16.

^d^ All but one patient received steroids as 1st line treatment.

^e^ Defined as mRS at last visit >2 or the need for a bilateral gait assistive device at the last visit. Median follow‐up duration for patients with outcome data and follow‐up duration data available was 23 months (*N* = 10).

### Cytokine/chemokine profiles and clinical correlations

**Figure 1:**
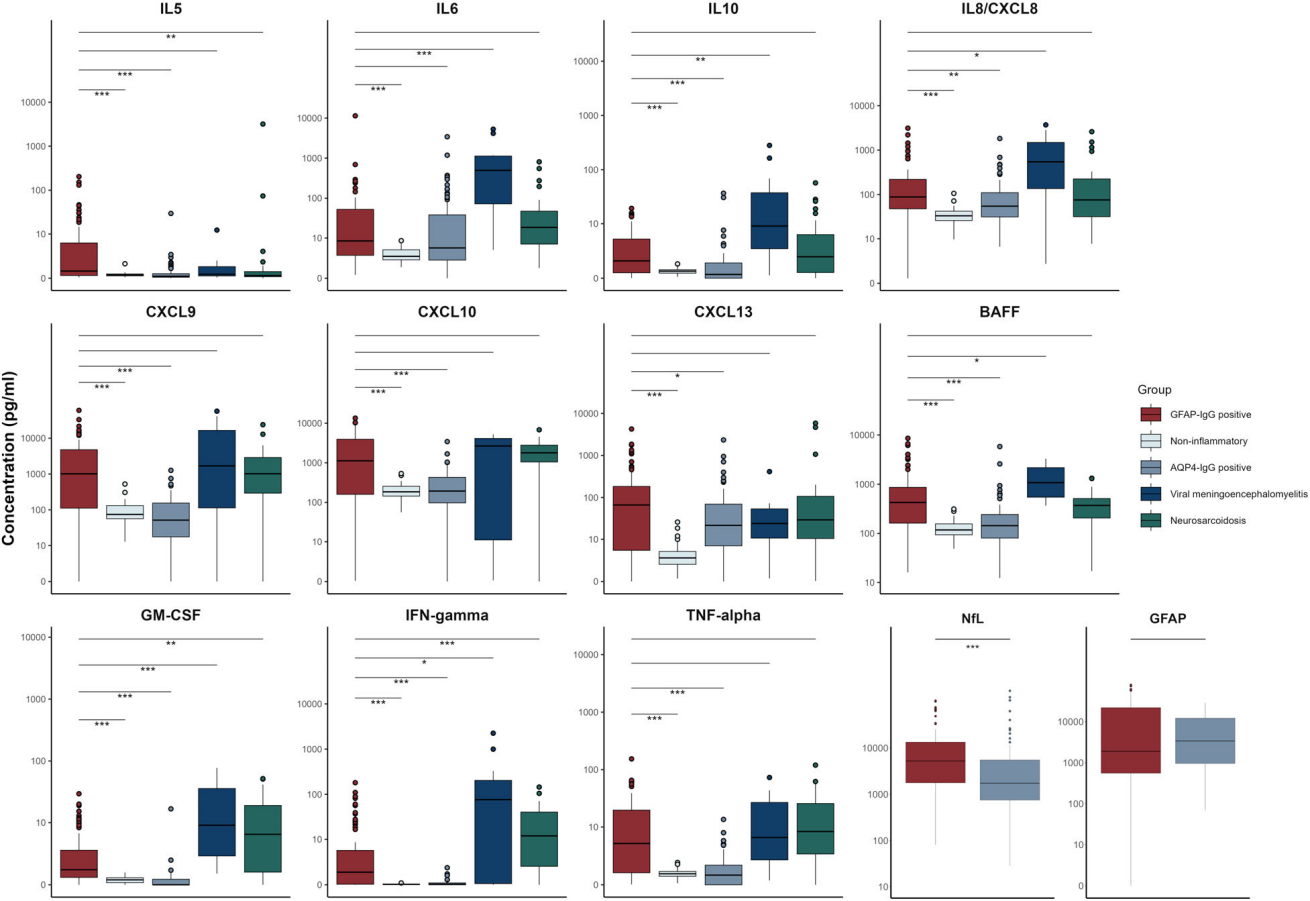
Cerebrospinal fluid cytokine, chemokine, and injury biomarker concentrations in GFAP‐IgG‐positive patients and controls. Only cytokines and chemokines significantly elevated in GFAP‐IgG‐positive patients in comparison to non‐inflammatory controls are presented. GFAP and NfL were compared between GFAP‐IgG‐positive and AQP4‐IgG‐positive cohorts. Significant differences between GFAP‐IgG‐positive patients and individual control groups (Wilcoxon rank‐sum test) are indicated by asterisks (*P < 0.05; **P < 0.01; ***P < 0.001). IFN, interferon; IL, interleukin; NfL, neurofilament light chain; TNF, tumor necrosis factor.

**Figure 2:**
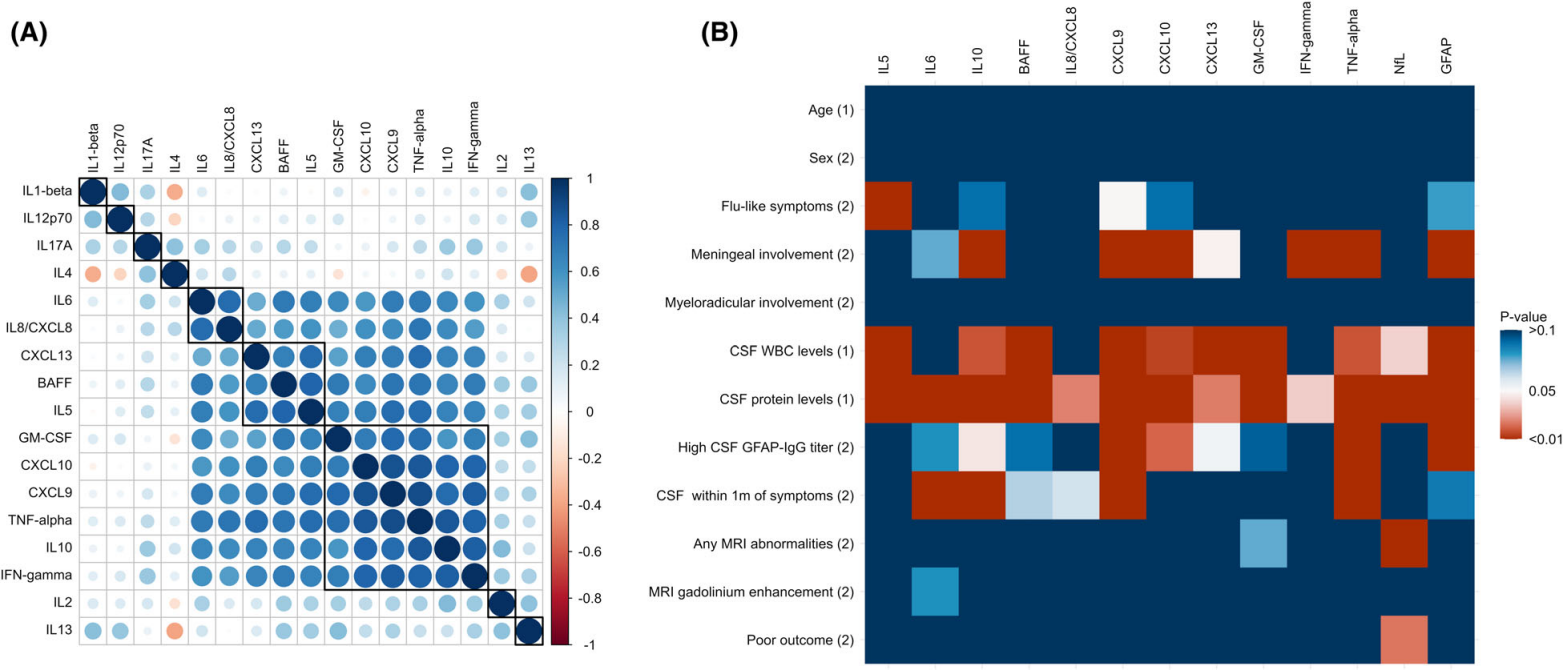
Clinical associations of elevated CSF analytes and inter‐analyte correlations among GFAP‐IgG‐positive patients. (A) Correlogram of Spearman's rank correlation coefficient matrix of GFAP‐IgG‐positive patients' analyte concentrations. (B) Clinical associations of elevated analytes and injury biomarkers. For cytokines and chemokines, analyte levels were treated as binary variables (elevated vs non‐elevated), and associations were tested with either the Wilcoxon rank‐sum test (1) or the Fisher exact test (2). GFAP and NfL were treated as continuous variables, and associations were tested with the Spearman correlation coefficient (for age) or the Wilcoxon rank‐sum test (for all other variables). CSF, cerebrospinal fluid; IFN, interferon; IL, interleukin; NfL, neurofilament light chain; TNF, tumor necrosis factor; WBC, white blood cell count.

Compared to non‐inflammatory controls (Fig. 1; Table S2), GFAP‐IgG‐positive patients had higher CSF concentrations of IL5, IL6, IL10, IL8/CXCL8, CXCL9, CXCL10, CXCL13, BAFF, GM‐CSF, IFN‐gamma, and TNF‐alpha (*P* < 0.01 for all); 77% had elevations in at least one of these. IFN‐gamma and BAFF were elevated most frequently (61% of patients for each), followed by TNF‐alpha (60%), GM‐CSF (58%), CXCL10 and CXCL13 (57% for each). Higher concentrations of IFN‐gamma, IL10, CXCL9, CXCL10, GM‐CSF, and TNF‐alpha clustered together (Fig. 2). Patients with atypical clinical presentations had lower IL5, IL10, CXCL13, TNF‐alpha, and GFAP concentrations (*P* < 0.05 for each).

CSF pleocytosis, high protein, high GFAP‐IgG titer (>1:16), and meningeal clinical signs correlated with different cytokine/chemokine elevations and biomarkers of neural injury (Fig. 2). CSF collected within 1 month of symptom onset was more likely to have elevated IL6, IL10, CXCL9, and TNF‐alpha (*P* < 0.01 for each). MRI abnormalities and poor outcomes were associated with higher NfL concentrations (*P* < 0.01 and *P* = 0.023, respectively; no significant age differences were noted between patients with/without MRI abnormalities or patients with/without poor outcomes). CSF collected prior to immunosuppressive therapy (*N* = 17) was more likely to have elevations of CXCL9, CXCL10, TNF‐alpha (*P* < 0.01 for each), IL10 (*P* = 0.018), IL5, and GM‐CSF (*P* < 0.05 for each) compared to CSF collected following treatment (*N* = 10).

Compared to AQP4‐IgG‐positive patients, the CSF of GFAP‐IgG‐positive patients had higher concentrations of IL5, IL10, IL8/CXCL8, CXCL9, CXCL10, BAFF, GM‐CSF, IFN‐gamma, and TNF‐alpha (*P* < 0.001 for each); GFAP concentrations did not differ significantly (*P* = 0.831), but GFAP‐IgG‐positive patients' CSF had higher NfL concentrations (*P* < 0.001).

Compared to CSF of viral meningoencephalitis controls, no analyte was significantly higher in GFAP‐IgG‐positive patients, but CSF of the viral meningoencephalitis patients had higher concentrations of IL1‐beta, IL2, IL6 (*P* < 0.001 for each), IL10 (*P* = 0.01), IL13, IL17A, IL8/CXCL8, BAFF, and IFN‐gamma (*P* < 0.03 for each).

Compared to neurosarcoidosis patients, the CSF of GFAP‐IgG‐positive patients had higher concentrations of IL5 (*P* = 0.001) and lower concentrations of GM‐CSF and IFN‐gamma (*P* < 0.01 for each).

## Discussion

This study reveals that GFAP‐IgG‐positive patients have elevated CSF cytokine levels attributable to macrophages, microglia, astrocytes, B‐cells, and T‐cells that are different from neurological diseases manifesting similarly and are reflective of the histopathological findings recently described in GFAP autoimmunity.^5^ The most prevalent histopathological phenotype reported is a lymphocytic parenchymal infiltrate, consisting mainly of T‐cells and reactive microglia/macrophages. Accordingly, our analysis showed clustering of IL10, CXCL9, CXC10, GM‐CSF, IFN‐gamma, and TNF‐alpha (Fig. 2).

IFN‐gamma, secreted mainly by T‐cells (Th1 and cytotoxic T‐cells) and natural killer cells, is a potent up‐regulator of CXCL9 and CXCL10 (also elevated in this study), which are T‐cell chemoattractants required for T‐cell mediated cytotoxicity and possibly microglial activation.^9^, ^10^ IFN‐gamma also upregulates major histocompatibility complex (MHC) class I expression^11^ which is upregulated on astrocytes in CNS lesions of GFAP‐IgG‐positive patients, rendering them susceptible to cytotoxic CD8^+^ T cell injury.^5^ Macrophage and microglia‐related cytokines were additionally elevated, including GM‐CSF (also produced by Th1, Th2, cytotoxic T‐cells, and potentially astrocytes), IL10 (also produced by regulatory T‐cells and Th2 cells), IL8/CXCL8, and TNF‐alpha (multiple other T‐cell sources and astrocytes).^12^, ^13^, ^14^, ^15^ TNF‐alpha drives granuloma formation,^12^ a phenotype described histopathologically and reflected in the elevations of cytokines pertinent to macrophages, microglia, and T‐cells. Elevations in BAFF and CXCL13 may reflect perivascular B‐cell infiltrates described in both histopathological phenotypes.^5^

IL5, a known regulator of eosinophil proliferation, secreted in the CNS by astrocytes and microglia   can induce microglial proliferation^16^ and possibly has a neuroprotective role in the setting of neural injury,^17^ was also elevated. IL6, a pleotropic cytokine produced by astrocytes, microglia, and various immune cells,^18^ showed the highest fold elevation. IL6 has a critical role in neuroinflammation, and blockade of its receptor is successfully applied in neuromyelitis optica spectrum disorder (NMOSD).^19^ While most patients with GFAP autoimmunity respond well to glucocorticosteroids, this treatment is associated with various side effects, and a subset of patients relapse; IL6 pathway blockade could be considered in this population.

GFAP protein CSF concentrations did not differ significantly between GFAP‐IgG‐positive patients and AQP4‐IgG‐positive patients, but NfL concentrations were higher in the former (*P* < 0.001), despite no significant difference in the two groups' age distribution. Previous pathological studies of GFAP autoimmunity have shown neuronal injury in some study patients.^5^, ^20^, ^21^, ^22^ NfL levels, reflecting such injury, could possibly have utility as a marker of disease severity in GFAP autoimmunity. This is further supported by the correlations noted in our clinical cohort between NfL levels and MRI abnormalities as well as poor outcomes. The similarity in GFAP concentrations among the two groups reflects the astrocytic injury, suggesting that GFAP may have utility as a biomarker of disease activity, similarly to AQP4‐IgG‐positive NMOSD. These findings have implications for future seronegative NMOSD diagnostic criteria, as GFAP elevations are not specific to NMOSD and could occur across diseases with astrocytic damage.

Limitations of this study are its retrospective nature and potential analyte degradation due to storage. Nevertheless, cytokines are relatively stable in CSF and the non‐inflammatory control samples used to establish reference range were rapidly frozen after collection.^8^ Additionally, only 30% of samples were known to be collected prior to immunotherapy, leading to potential underestimation of cytokine elevations. Lastly, the paucity of available clinical data (available for 56/98 patients) limited the analysis of clinical correlations and the lack of clinical data for the AQP4‐IgG‐positive cohort hinders GFAP and NfL data interpretation as these might have different dynamics in relation to clinical activity.^23^

Overall, the CSF cytokine profiles identified in GFAP‐IgG‐positive patients complement the CNS histopathological findings, inform the pathogenesis of GFAP autoimmunity, and suggest therapeutic targets.

## Author Contributions

Y.S. and A.Z. contributed to the conception and design of the study; all authors contributed to the acquisition and analysis of data; Y.S., G.M., and A.Z. contributed to drafting the text or preparing the figures.

## Conflicts of Interest

Y.S., G.M., B.Y., M.T., J.R.M., and D.D. report no relevant conflicts of interest. V.L. shares in royalties paid to Mayo Clinic from licensing of AQP4‐IgG diagnostic tests performed outside Mayo Clinic. V.L. and A.M.K. have a U.S. patent filed for methods and materials for identifying and treating autoimmune GFAP astrocytopathy. E.P.F. has served on advisory boards for Roche/Genentech, and he is a site principal investigator and a member of the steering committee for a clinical trial of satralizumab for relapsing myelin oligodendrocyte glycoprotein antibody‐associated disease run by Roche/Genentech. I.V. has received personal compensation for serving as an employee of F. Hoffmann‐La Roche Ltd (Roche) and has stock in Roche. S.J.P. has received personal compensation for serving as a consultant and for serving on scientific advisory boards or data safety monitoring boards for Roche/Genentech; he also received research support from Roche/Genentech. A.Z. has received research funding from Roche and the Mayo Clinic Center for MS and Autoimmune Neurology relevant to this work.

## Supporting information


Data S1:

## Data Availability

The data that support the findings of this study are available from the corresponding author upon reasonable request.
