# Supplementary material for: CSF cytokine, chemokine and injury biomarker profile of glial fibrillary acidic protein (GFAP) autoimmunity
Source: Ann Clin Transl Neurol. 2025 Jan 27;12(4):855–60. doi: 10.1002/acn3.52305 (PMC12040519; doi:10.1002/acn3.52305)
Supplement: Supplementary file 1 — Data S1: [file ACN3-12-855-s001.docx]

**SUPPLEMENTARY MATERIAL**

**CONTENTS**

Table S1. Baseline characteristics of disease cohorts

Table S2. Comparison of CSF analyte levels between GFAP-IgG-positive patients and controls

| **Table S1. Baseline characteristics of disease cohorts** | | | | | | | | | |
| --- | --- | --- | --- | --- | --- | --- | --- | --- | --- |
|  | **GFAP-IgG positive patients (N=98)** | **Non-inflammatory controls^a^ (N=42)** | **P-value ^b^** | **AQP4-IgG positive patients^c^ (N=83)** | **P-value ^b^** | **Viral Meningoencephalomyelitis^d^**  **(N=13)** | **P-value ^b^** | **Neurosarcoidosis^c^ (N=32)** | **P-value ^b^** |
| Age at collection, median (range), years | 43  (4-80) | 74  (21-87) | **< 0.001^e^** | 47  (2-90) | 0.503^e^ | 68  (2-85) | **0.023^e^** | 59  (19–81) | **0.026^e^** |
| Female sex (%) | 51 (52) | 17 (40.5) | 0.269^f^ | 70 (84) | **< 0.001^f^** | 8 (61.5) | 0.568^f^ | 19/32 (59) | 0.543 |
| CSF pleocytosis present (%) | 45/55 (82) | 0/42 (0) | **< 0.001^f^** | NA | NA | 13/13 (100) | 0.189^f^ | 27/32 (84) | 1.0^f^ |
| ^a^ Diagnoses of non-inflammatory controls included: 35 NPH; 2 IIH; 2 FND; 1 congenital hydrocephalus, 1 ex vacuo ventriculomegaly, 1 idiopathic peripheral neuropathy. Three NPH patients had concomitant diagnosis of neurodegenerative conditions (either Alzheimer’s disease or Dementia with Lewy bodies), one additional NPH patient was also diagnosed with primary lateral sclerosis.  ^b^ compared to GFAP-IgG positive patients.  ^c^ Previously reported (1).  ^d^ Diagnoses of Viral Meningoencephalomyelitis included: 5 WNV; 4 HSV1; 3 VZV; 1 Powassan virus.  ^e^ Wilcoxon rank sum test.  ^f^ Fisher’s exact test.  * All non-inflammatory control samples were centrifuged, processed, and stored at -80oC within 2 hours of collection while the rest were retrospectively identified and could have stayed at 4oC for 30 days before freezing, potentially affecting analyte concentrations.  AQP4=aquaporin 4; CNS=central nervous system; CSF=cerebrospinal fluid; h=hours; FND= functional neurological disorder; HSV1 = Herpes Simplex 1 virus; IIH= idiopathic intracranial hypertension; IST=immunosuppressive therapy; NA=missing value or non-applicable; NPH= normal pressure hydrocephalus; VZV= Varicella Zoster virus; WBC=white blood cells; WNV= West Nile virus. | | | | | | | | | |

| **Table S2. Comparison of CSF analyte concentrations between GFAP-IgG-positive patients and controls** | | | | | | | | | | | |
| --- | --- | --- | --- | --- | --- | --- | --- | --- | --- | --- | --- |
|  | **GFAP-IgG-positive patients (N=98)** | | | **Non-inflammatory (N=42)** | | **AQP4-IgG-positive patients (N=83)^a^** | | **Viral meningoencephalitis (N=13)** | | **Neurosarcoidosis (n = 32)^a^** | |
| **Analyte** | **Analyte concentrations, median (range), pg/mL** | **Elevated (%)** | **Fold elevation^b^, median (range)** | **Analyte concentrations, median (range), pg/mL** | **P value^c^** | **Analyte concentrations, median (range), pg/mL** | **P value^c^** | **Analyte concentrations, median (range), pg/mL** | **P value^c^** | **Analyte concentrations, median (range), pg/mL** | **P value^c^** |
| **IL1-beta** | 0.2 (0 - 11.8) | 7/96 (7) | NA^d^ | 0.3 (0.0-0.8) | NA^d^ | 0.0 (0.0-40.2) | NA^d^ | 0.8 (0.2-7.4) | <0.001 | 0.4 (0-2.8) | NA^e^ |
| **IL2** | 0.2 (0 - 1.8) | 15/97 (16) | NA^d, f^ | 0.2 (0.1-0.5) | NA^d, f^ | 0.0 (0.0-2.2) | NA^d, e^ | 1.0 (0.4-24.2) | <0.001 | 0.4 (0.1-4.6) | NA ^f^ |
| **IL4** | 0.1 (0-0.5) | 0/98 (0) | NA^d, f^ | 0.0 (0.0-0.2) | NA^d, f^ | 0.0 (0.0-5.7) | NA^d, e^ | 0.1 (0.0-0.7) | 0.75 | 0 (0-0.1) | NA^d, f^ |
| **IL5** | 0.8 (0.1-203.7) | 38/98 (39) | 6.0 (1.1-122.7) | 0.3 (0.1-1.7) | <0.001 | 0.2 (0.0-29.6) | <0.001 | 0.4 (0.1-12.3) | 0.156 | 0.3 (0-3184) | 0.001 |
| **IL6** | 8.4 (0.4-11399.0) | 47/94 (50) | 66.9 (10.8-14248.8) | 3.2 (1.4-8.6) | <0.001 | 5.5 (0.0-3423.0) | 0.337 | 493.5 (4.9-5274.5) | <0.001 | 18.6 (1.2-808) | 0.123 |
| **IL10** | 1.6 (0.0-19.4) | 53/98 (54) | 3.6 (1.1-15.0) | 0.6 (0.1-1.3) | <0.001 | 0.3(0.0-36.8) | <0.001 | 9.0 (0.2-280.5) | 0.001 | 2.1 (0-57.1) | 0.349 |
| **IL12p70** | 0.1 (0.0-1.2) | 1/90 (1) | NA^d, f^ | 0.4 (0.0-1.0) | NA^d, f^ | 0.0 (0.0-71.2) | NA^d, f^ | 0.1 (0.0-3.3) | 0.419 | 0.2 (0-0.9) | NA^f^ |
| **IL13** | 0.1 (0.0-17.5) | 12/97 (12) | NA^d, f^ | 0.0 (0.0-2.1) | NA^d, f^ | 0.0 (0.0-918.0) | NA^d, f^ | 1.6 (0.0-529.5) | 0.017 | 0 (0-15.2) | NA^d, f^ |
| **IL17A** | 0.9 (0.0-81.6) | 15/93 (16) | NA^d, f^ | 0.7 (0.0-1.8) | NA^d, f^ | 0.0 (0.0-8.6) | NA^d, f^ | 1.8 (0.0-5.9) | 0.011 | 0.2 (0-2.9) | NA^d, f^ |
| **BAFF** | 426.7 (16.2-8566.0) | 59/97 (61) | 2.4 (1.1-27.3) | 118.0 (49.0-314.0) | <0.001 | 144.0 (12.4-5816.5) | 0.001 | 1074.7 (366.7-3292.7) | 0.014 | 371 (17.2-1324) | 0.265 |
| **IL8/CXCL8** | 88.1 (0.5-3124.7) | 43/98 (44) | 2.2 (1.0-29.5) | 33.1 (9.7-106.0) | <0.001 | 54.7 (6.5-1826.0) | 0.004 | 545.7 (2.4-3704.7) | 0.02 | 75.6 (7.7-2602) | 0.611 |
| **CXCL9** | 1013.5 (0.0-59941.5) | 53/98 (54) | 8.1 (1.2-114.2) | 74.7 (12.9-525.0) | <0.001 | 51.7 (0.0-1265.0) | <0.001 | 1686.5 (0.0-56789.0) | 0.492 | 1019 (0-23848) | 0.886 |
| **CXCL10** | 1124.5 (0.1- 13468.0) | 54/95 (57) | 6.9 (1.1-25.1) | 185.0 (56.1-537.0) | <0.001 | 193.0 (0.0-3433.0) | <0.001 | 2650.0 (0.1-5260.0) | 0.708 | 1789.8 (0-6819.5) | 0.769 |
| **CXCL13** | 66.3 (0.0-4261.0) | 55/97 (57) | 6.7 (1.1-163.9) | 3.4 (0.3-26.0) | <0.001 | 21.6 (0.0 - 2340.0) | 0.042 | 24.0 (0.3-413.0) | 0.22 | 29.3 (0.1-5859) | 0.611 |
| **GM-CSF** | 1.2 (0.0-29.5) | 55/95 (58) | 3.2 (1.1-30.9) | 0.4 (0.0-1.0) | <0.001 | 0.0 (0.0-16.8) | <0.001 | 9.1 (0.9-77.3) | <0.001 | 6.4 (0-51.9) | 0.001 |
| **IFN-gamma** | 1.4 (0.0-181.0) | 57/93 (61) | 19.9 (1.1-957.7) | 0.0 (0.0-0.2) | <0.001 | 0.1 (0.0-2.0) | <0.001 | 76.5 (0.0-2249.0) | 0.014 | 12 (0-145) | < 0.001 |
| **TNF-alpha** | 5.0 (0.0-154.0) | 57/95 (60) | 7.6 (1.0-75.5) | 0.9 (0.1-2.0) | <0.001 | 0.8 (0.0-13.6) | <0.001 | 6.5 (0.3-73.2) | 0.686 | 8.3 (0-120) | 0.317 |
| **GFAP** | 1894.5 (0.0 - 78889.3) | NA | NA | NA | NA | 3392.5 (69.0 - 28874.0)^g^ | 0.831 | NA | NA | NA | NA |
| **NfL** | 5256.2 (80.8 - 105098.0) | NA | NA | NA | NA | 1741.0 (28.5 - 172055.0) | <0.001 | NA | NA | NA | NA |
| ^a^ Previously reported (1).  ^b^ Among patients with elevated analyte levels, compared to the maximum value of non-inflammatory controls.  ^c^ Compared to GFAP-IgG positive patients (Wilcoxon rank sum test).  ^d^ Less than 20% of GFAP-IgG positive patients with abnormal values.  ^e^ levels for neurosarcoidosis patients were not significantly elevated compared to non-inflammatory controls (1) even though elevated in comparison to GFAP-IgG positive patients; given loe levels it was noted as NA compared to GFAP-IgG positive patients.  ^f^ Highest median values measured were bellow LLOQ.  ^g^ performed for patients with sufficient available CSF, N=48.  NA=not applicable. | | | | | | | | | | | |

1. Mangioris G, Pittock SJ, Yang B, Fryer JP, Harmsen WS, Dubey D, et al. Cerebrospinal Fluid Cytokine and Chemokine Profiles in Central Nervous System Sarcoidosis: Diagnostic and Immunopathologic Insights. Ann Neurol. 2024.
